# Neutralizing Monoclonal Antibodies Inhibit SARS-CoV-2 Infection through Blocking Membrane Fusion

**DOI:** 10.1128/spectrum.01814-21

**Published:** 2022-03-16

**Authors:** Chia-Jung Li, Tai-Ling Chao, Ting-Yu Chang, Chia-Chun Hsiao, De-Chao Lu, Yi-Wei Chiang, Guan-Chun Lai, Ya-Min Tsai, Jun-Tung Fang, Siman Ieong, Jann-Tay Wang, Sui-Yuan Chang, Shih-Chung Chang

**Affiliations:** a Department of Biochemical Science and Technology, College of Life Science, National Taiwan University, Taipei, Taiwan; b Department of Clinical Laboratory Sciences and Medical Biotechnology, College of Medicine, National Taiwan University, Taipei, Taiwan; c Genomics Research Center, Academia Sinica, Taipei, Taiwan; d Department of Internal Medicine, National Taiwan University Hospital, Taipei, Taiwan; e Department of Laboratory Medicine, National Taiwan University Hospital, College of Medicine, National Taiwan University, Taipei, Taiwan; f Center of Biotechnology, National Taiwan University, Taipei, Taiwan; Shandong First Medical University

**Keywords:** neutralizing antibodies, SARS-CoV-2, spike (S) protein, S2 subunit, membrane fusion

## Abstract

Most of SARS-CoV-2 neutralizing antibodies (nAbs) targeted the receptor binding domain (RBD) of the SARS-CoV-2 spike (S) protein. However, mutations at RBD sequences found in the emerging SARS-CoV-2 variants greatly reduced the effectiveness of nAbs. Here we showed that four nAbs, S2-4D, S2-5D, S2-8D, and S2-4A, which recognized a conserved epitope in the S2 subunit of the S protein, can inhibit SARS-CoV-2 infection through blocking the S protein-mediated membrane fusion. Notably, these four nAbs exhibited broadly neutralizing activity against SARS-CoV-2 Alpha, Gamma, Delta, and Epsilon variants. Antisera collected from mice immunized with the identified epitope peptides of these four nAbs also exhibited potent virus neutralizing activity. Discovery of the S2-specific nAbs and their unique antigenic epitopes paves a new path for development of COVID-19 therapeutics and vaccines.

**IMPORTANCE** The spike (S) protein on the surface of SARS-CoV-2 mediates receptor binding and virus-host cell membrane fusion during virus entry. Many neutralizing antibodies (nAbs), which targeted the receptor binding domain (RBD) of S protein, lost the neutralizing activity against the newly emerging SARS-CoV-2 variants with sequence mutations at the RBD. In contrast, the nAb against the highly conserved S2 subunit, which plays the key role in virus–host cell membrane fusion, was poorly discovered. We showed that four S2-specific nAbs, S2-4D, S2-5D, S2-8D, and S2-4A, inhibited SARS-CoV-2 infection through blocking the S protein-mediated membrane fusion. These nAbs exhibited broadly neutralizing activity against Alpha, Gamma, Delta, and Epsilon variants. Antisera induced by the identified epitope peptides also possessed potent neutralizing activity. This work not only unveiled the S2-specific nAbs but also discovered an immunodominant epitope in the S2 subunit that can be rationally designed as the broad-spectrum vaccine against the SARS-like coronaviruses.

## INTRODUCTION

The ongoing coronavirus disease 2019 (COVID-19) outbreak, which is caused by severe acute respiratory syndrome coronavirus 2 (SARS-CoV-2), is a threatening crisis to global health and economy. Development of antiviral drug and vaccine will save human lives and help combat the COVID-19 pandemic. The primary vaccine target is the trimeric spike (S) protein on the surface of SARS-CoV-2, that plays an essential role in virus entry (1, 2). The S protein consists of an N-terminal S1 subunit that mediates receptor binding, and a C-terminal S2 subunit that mediates virus–host cell membrane fusion (1). The S1 subunit has two major structural elements, the N-terminal domain (NTD) and the receptor binding domain (RBD), through which virus can bind to the angiotensin-converting enzyme 2 (ACE2) receptor on its host cells (3–7). The S2 subunit contains fusion peptide, heptad repeats 1 and 2 (HR1 and HR2), a central helix, a connector domain, a transmembrane domain, and a cytoplasmic tail (2, 8). After binding of the RBD to the ACE2 receptor, the sequential cleavages of the S protein at the S1/S2 and the S2′ cleavage sites trigger an irreversible conformational change in the S2 subunit that subsequently initiates membrane fusion with the host cell (1, 4, 9, 10).

Monoclonal antibodies (mAbs) with strong virus neutralizing activity are potential candidates for development as prophylactic or therapeutic agents against SARS-CoV-2 infection. Many SARS-CoV-2 neutralizing antibodies (nAbs) can block RBD binding to ACE2 and exhibit very high potency in inhibiting SARS-CoV-2 infection (11–24). Some of these antibodies have been reported with prophylactic or therapeutic functionality against SARS-CoV-2 infection in animal models (15, 20, 22, 25). Other groups of nAbs recognized the molecular determinants in the NTD (15, 26) or the quaternary epitopes that overlap with the domains at the top of the S protein (15). Detailed characterization of this diverse collection of nAbs has provided evidence that most of the SARS-CoV-2 nAbs are directed against the S1 subunit of the S protein, leading to the blockage of virus interaction with the ACE2 receptor.

The S2 subunits of the S proteins derived from SARS-CoV and SARS-CoV-2 share ∼90% sequence identity and also contain neutralizing epitopes (27–29). However, the nAbs against the S2 subunit of SARS-CoV-2 were poorly defined and investigated. To generate the S2-specific MAb for recognition of the SARS-CoV-2 S protein, the recombinant S2 subunit expressed in Expi293F cells was utilized as the antigen for immunization of mice. Five mAbs, S2-10H, S2-4D, S2-5D, S2-8D, and S2-8A, were generated through the conventional hybridoma technology, and selected for further biochemical and functional characterization. Among them, S2-4D, S2-5D, and S2-8D exhibited great binding specificity with SARS-CoV-2 S protein and S2 subunit, and also performed potent neutralizing activity against SARS-CoV-2 infection in Vero E6 cells. The antigenic determinants in the S2 subunit recognized by S2-4D, S2-5D, and S2-8D were further analyzed to study the molecular basis for blocking the S protein-mediated virus–host cell membrane fusion by these unique mAbs. More importantly, the antisera induced by immunization of mice with the antigenic peptides recognized by S2-4D, S2-5D, and S2-8D also exhibited potent neutralizing activity against diverse SARS-CoV-2 variants. This work not only unveiled the S2-specific SARS-CoV-2 nAbs but also identified an immunodominant epitope in the S2 subunit that can induce broadly neutralizing antibodies against the emerging SARS-like coronaviruses.

## RESULTS

### The SARS-CoV-2 S2-specific mAbs.

**FIG 1:**
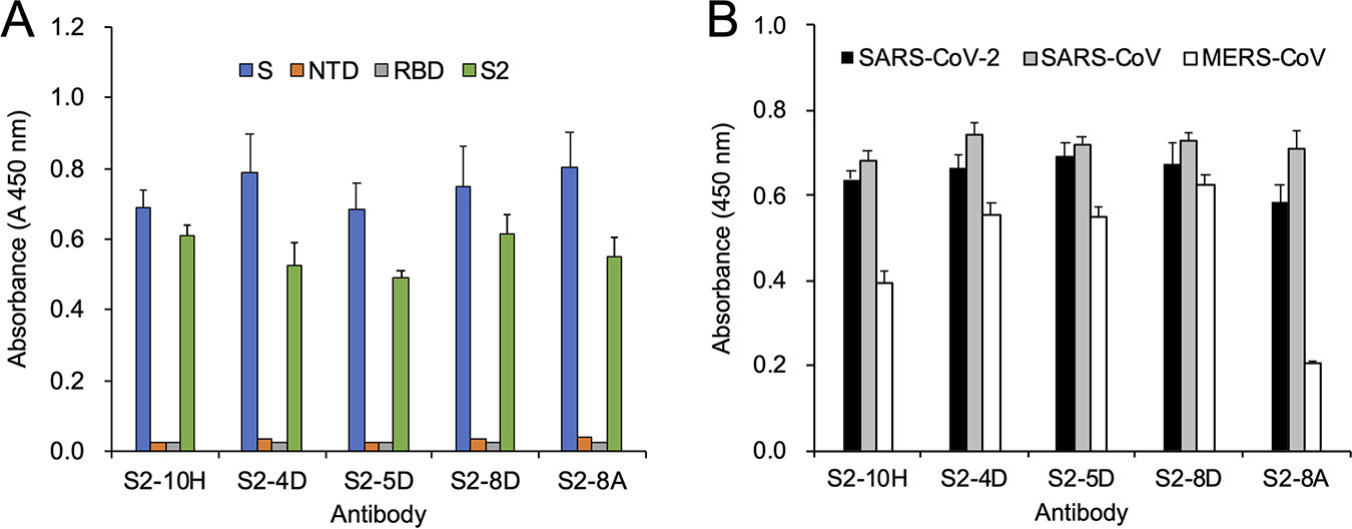
The binding specificity of anti-S2 mAbs. (A) The recombinant SARS-CoV-2 S, NTD, RBD, and S2 proteins or (B) the recombinant S proteins of SARS-CoV-2, SARS-CoV, and MERS-CoV were utilized as the antigens in the ELISA experiments for analyzing the binding specificity of mAbs S2-10H, S2-4D, S2-5D, S2-8D, and S2-8A. Absorbance was measured at 450 nm by a spectrophotometer. Data are presented as means ± SD of three biological replicates (*n* = 3).

To generate the S2-specific MAb for recognition of the SARS-CoV-2 S protein, the recombinant S2 subunit expressed in Expi293F cells was utilized to immunize mice. Five mAbs, S2-10H, S2-4D, S2-5D, S2-8D, and S2-8A, were generated through the conventional hybridoma technology, and selected for further biochemical characterization. These five mAbs showed great binding specificity to SARS-CoV-2 S protein and S2 subunit without exhibiting cross-reactivity to the NTD and the RBD (Fig. 1A). These five mAbs also exhibited strong cross-reactivity to the SARS-CoV S proteins (Fig. 1B), implying that the antibody binding epitopes recognized by these mAbs might be highly conserved between SARS-CoV-2 and SARS-CoV S proteins. Additionally, S2-10H, S2-4D, S2-5D, S2-8D, and S2-8A exhibited different levels of cross-reactivity with the Middle East respiratory syndrome coronavirus (MERS-CoV) S proteins (Fig. 1B).

### Inhibition of S protein-mediated membrane fusion and SARS-CoV-2 infection by S2-4D, S2-5D, and S2-8D.

**FIG 2:**
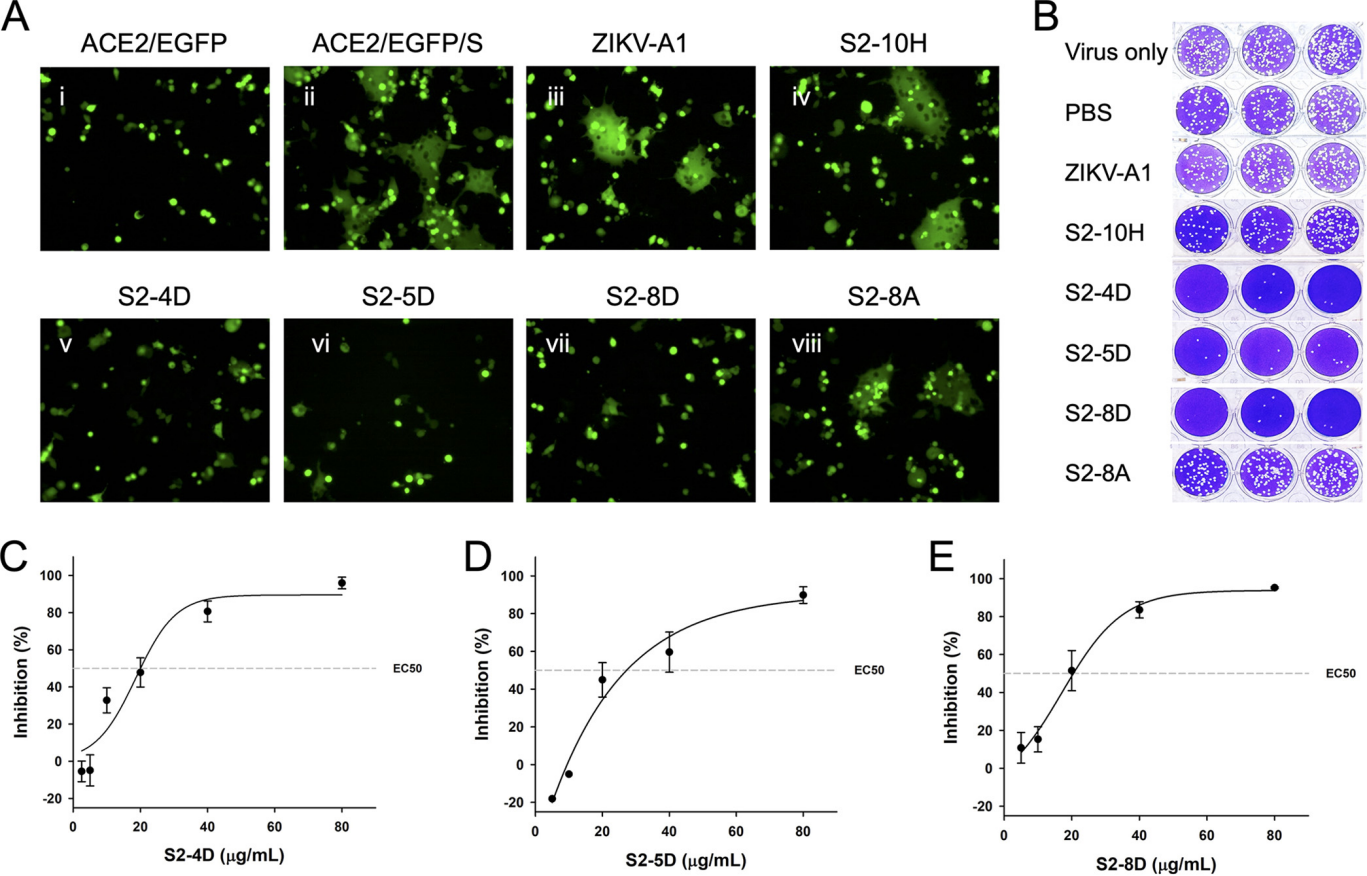
The functionality of anti-S2 mAbs on inhibition of membrane fusion and virus infection. (A) For performing the S protein-mediated syncytium formation assay, ZIKV-A1 (isotype control antibody), S2-10H, S2-4D, S2-5D, S2-8D, or S2-8A (100 μg/mL for each) was preincubated with S protein-transfected 293T cells individually, before being added to the EGFP-transfected ACE2-293T cells. After 16 h of coculture, the syncytium formation was observed by fluorescence microscopy. (B) For performing the three biological replicates (*n* = 3) of plaque reduction assay, the indicated antibody (100 μg/mL) was incubated with the SARS-CoV-2 wild-type strain in the presence of 8 μg/mL TPCK-trypsin of DMEM for 1 h at 37°C. Antibody-virus mixtures (200 μL/well) were subsequently added to the Vero E6 cell monolayers for one additional hour in 24-well plates. Five to 7 days later, cells were fixed with formaldehyde and stained with crystal violet. (C–E) The 50% of neutralization (EC50) of SARS-CoV-2 infection by mAbs S2-4D, S2-5D, and S2-8D were determined by using a series of diluted MAb solutions (80, 40, 20, 10, 5 μg/mL) in the plaque reduction assay. Data are presented as means ± SD of three biological replicates (*n* =3), and further graphed by linear regression.

Since the S2 subunit plays the key role in mediating virus–host cell membrane fusion during SARS-CoV-2 infection, the S protein-mediated syncytium formation assay was performed to examine whether S2-10H, S2-4D, S2-5D, S2-8D, and S2-8A could block syncytium formation. As shown in Fig. 2A, S2-4D, S2-5D, and S2-8D could significantly inhibit the syncytium formation (Fig. 2A v–vii), while only moderate inhibition was observed in the presence of S2-8A (Fig. 2A, viii). To be noted, S2-10H showed no inhibition of syncytium, like the isotype control antibody ZIKV-A1 (30) (Fig. 2A, iii and iv).

To further determine whether S2-10H, S2-4D, S2-5D, S2-8D, and S2-8A could inhibit SARS-CoV-2 infection in Vero E6 cells, the plaque reduction assay was conducted in the Biosafety level 3 (BSL-3) laboratory. Concordant with the observation in the S protein-mediated membrane fusion assay, S2-4D, S2-5D, and S2-8D can efficiently inhibit SARS-CoV-2 infection (Fig. 2B) with EC50 values of 20–30 μg/mL (Fig. 2C to E). The S2-10H only exhibited slight inhibition activity, while S2-8A and the isotype control antibody ZIKV-A1 cannot prevent SARS-CoV-2 infection (Fig. 2B).

### Epitope mapping of SARS-CoV-2 S2-specific mAbs.

**FIG 3:**
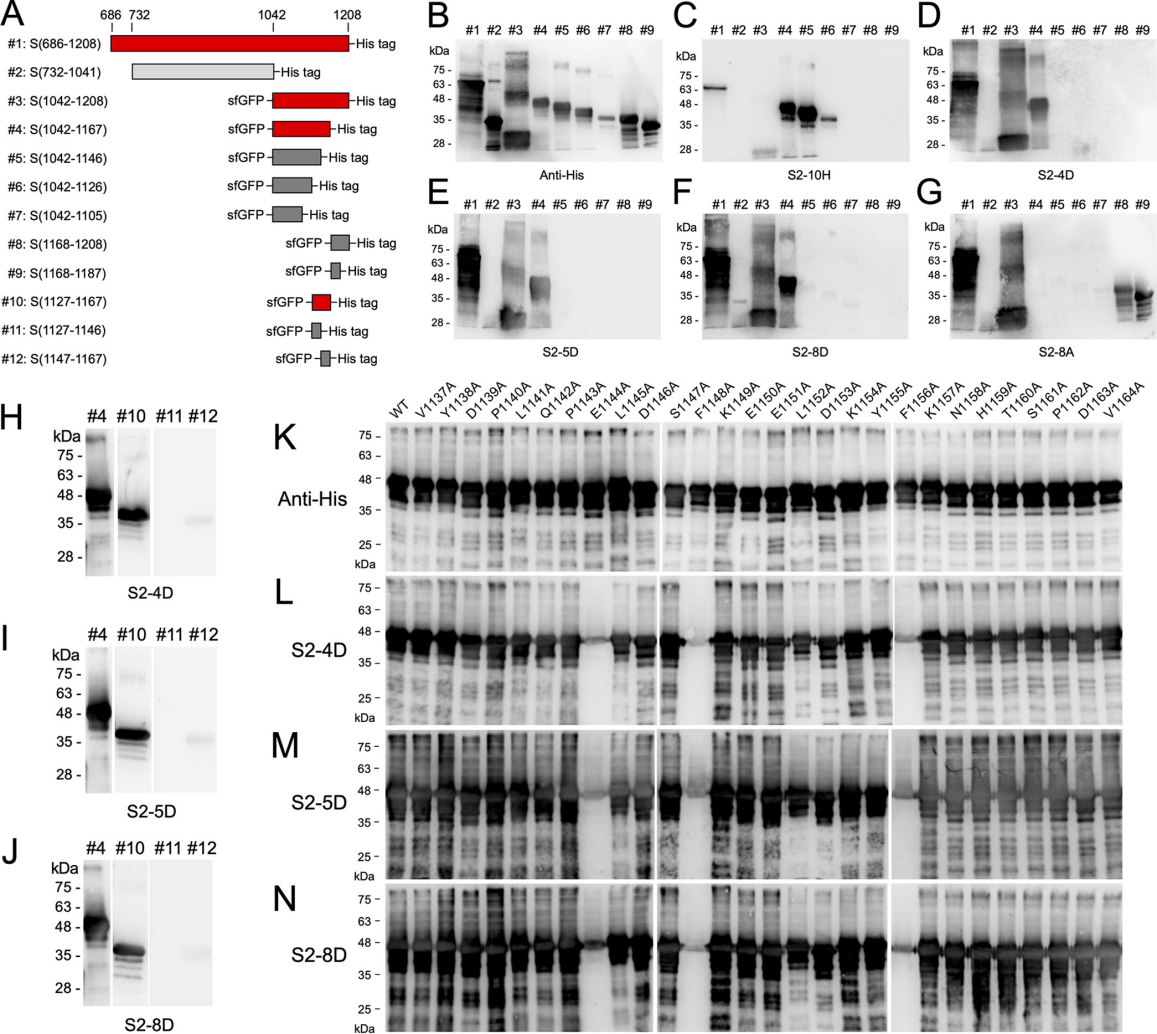
Analysis of the binding epitopes of anti-S2 mAbs. (A) The schematic diagram illustrated the design of the S2 truncation fragments. Fragments #1 and #2 contain a C-terminal His-tag. Fragments #3–12 contain an N-terminal sfGFP and a C-terminal His-tag. For easy reading of the WB results, the fragments that can be detected by mAbs S2-4D, S2-5D, and S2-8D are marked in red. (B–J) The binding activities of the control anti-His tag antibody, and mAbs S2-4D, S2-5D, and S2-8D against the S2 truncation fragments #1–12 were determined by WB analysis. (K–N) The recombinant sfGFP-S(1042-1167) (fragment #4) mutants with a series of individual alanine substitutions at residues 1137–1164 were expressed in E. coli, and the bacterial cell lysates (6 μg) were subjected to SDS-PAGE and WB analysis with mAbs S2-4D, S2-5D, and S2-8D to identify the critical antigenic determinants.

Although S2-10H, S2-4D, S2-5D, S2-8D, and S2-8A have been characterized as S2-specific mAbs (Fig. 1), the functionality of S2-10H and S2-8A was different from that of S2-4D, S2-5D, and S2-8D in the aspect of inhibiting SARS-CoV-2 infection (Fig. 2). It was proposed that these mAbs might recognize different binding epitopes in the S2 subunit. Therefore, a total of 12 fragments spanning the S2 subunit were generated for epitope mapping (Fig. 3A). Among them, fragments #3–12 were expressed with an N-terminal sfGFP fusion tag to increase the protein solubility (31). The S2-10H can recognize fragments #3: S(1042-1208), #4: S(1042-1167), #5: S(1042-1146), and #6: S(1042-1126), but not fragment #7: S(1042-1105), implicating that the binding epitope of S2-10H is located at the region of S(1106-1126) (Fig. 3C). As shown in Fig. 3D–F, S2-4D, S2-5D, and S2-8D can recognize fragments #3: S(1042-1208) and #4: S(1042-1167), but not fragment #5: S(1042-1146), suggesting that the binding epitopes for these three mAbs might be located at the region of S(1147-1167). The binding patterns of S2-8A were different from other S2-specific mAbs, in that it can only recognize fragments #3: S(1042-1208), #8: S(1168-1208), and #9: S(1168-1187). Therefore, the binding epitope of MAb S2-8A is likely located at the region of S(1168-1187) (Fig. 3G).

To further determine the potential binding epitopes of S2-4D, S2-5D, and S2-8D, three shorter fragments, #10: S(1127-1167), #11: S(1127-1146), and #12: S(1147-1167) were generated for epitope mapping. Surprisingly, all of S2-4D, S2-5D, and S2-8D performed a strong binding to fragment #10: S(1127-1167), but exhibited no binding to fragment #11: S(1127-1146) or a very weak binding to fragment #12: S(1147-1167) (Fig. 3H to J), implying that the binding epitopes of S2-4D, S2-5D, and S2-8D might include the upstream and downstream residues of S(1146-1147).

To identify the critical antigenic determinants of S2-4D, S2-5D, and S2-8D, the amino acid residues 1137–1164 of sfGFP-S(1042-1167) were substituted with alanine individually to generate a series of sfGFP-S(1042-1167) mutants for Western blotting (WB) analysis with the control anti-His tag antibody (Fig. 3K), S2-4D (Fig. 3L), S2-5D (Fig. 3M), and S2-8D (Fig. 3N). The results clearly showed that residues E1144, F1148, and F1156 are the critical antigenic determinants for S2-4D, S2-5D, and S2-8D recognition, since replacement of these residues with alanine abrogated the binding of these mAbs to sfGFP-S(1042-1167) (Fig. 3L to N). Furthermore, by using less amounts of sfGFP-S(1042-1167) mutants on the SDS-PAGE to perform the WB analysis, we observed that the L1152A mutation might also moderately reduce the binding efficiency of S2-4D, S2-5D, and S2-8D, suggesting that L1152 could be also involved in the interaction with these three mAbs (Fig. S1 in the supplemental material).

### The functionality of the antisera induced by the defined epitope peptides of S2-4D, S2-5D, and S2-8D.

**FIG 4:**
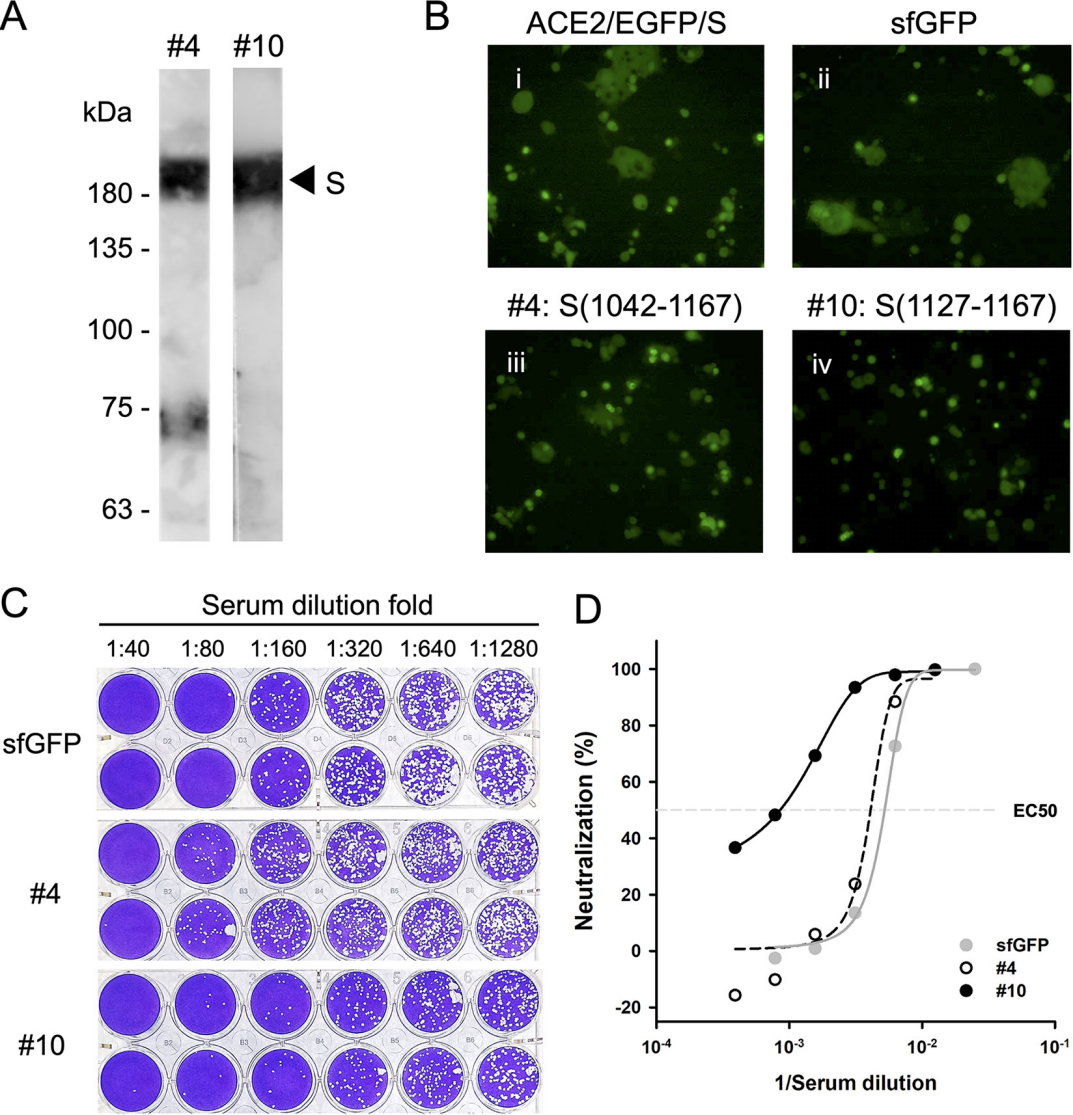
Functional analysis of the antisera raised by the binding epitope of S2-4D, S2-5D, and S2-8D. (A) Detection of the recombinant SARS-CoV-2 S protein expressed in Expi293F cells by WB analysis using the antisera raised by immunization of mice with sfGFP-S(1042-1208) (fragment #4) and sfGFP-S(1127-1167) (fragment #10). (B) Inhibition of S protein-mediated syncytium formation was performed using 100 μg/mL of antisera (i, no antiserum; ii, sfGFP group; iii, fragment #4 group; iv, fragment #10 group). (C) The plaque reduction assay with the SARS-CoV-2 wild-type strain was performed using a series of diluted antisera raised by immunization of mice with sfGFP, fragment #4, and fragment #10. Data are two biological replicates (*n* = 2). (D) The results (shown as means of neutralization percentage of two biological replicates) of the SARS-CoV-2 plaque reduction assay in C are plotted with the reciprocal values of the serum dilution fold, and further graphed by linear regression. The EC50 is noted with a dashed line. Gray solid circles represent the results from the sfGFP group. White open circles represent the results from the fragment #4 group. Black solid circles represent the results from the fragment #10 group.

To determine whether the identified binding epitope of S2-4D, S2-5D, and S2-8D can be used as a potential vaccine candidate, sfGFP-S(1042-1167) and sfGFP-S(1127-1167) were expressed in Escherichia coli (E. coli) and purified for antigen immunization in mice. The antisera were collected for analyzing the specificity and functionality. The results showed that antisera induced by sfGFP-S(1042-1167) and sfGFP-S(1127-1167) can bind to the recombinant SARS-CoV-2 S protein expressed in Expi293F cells (Fig. 4A), indicating that the specific antisera have been successfully induced. As shown in Fig. 4B, the sfGFP-S(1127-1167) group exhibited strong neutralizing activity for inhibiting the S protein-mediated syncytium formation (Fig. 4B, iv), while the sfGFP-S(1042-1167) group only had minor neutralizing activity (Fig. 4B, iii). The ability of these antisera to neutralize SARS-CoV-2 infection was also examined by using the plaque reduction assay (Fig. 4C). Concordant with the results observed for inhibition of syncytium formation, the sfGFP-S(1127-1167) group exhibited the strongest neutralization activity since its serum dilution fold for reaching EC50 is five folds diluted from that of the sfGFP group or the sfGFP-S(1042-1167) group (Fig. 4C and D).

**FIG 5:**
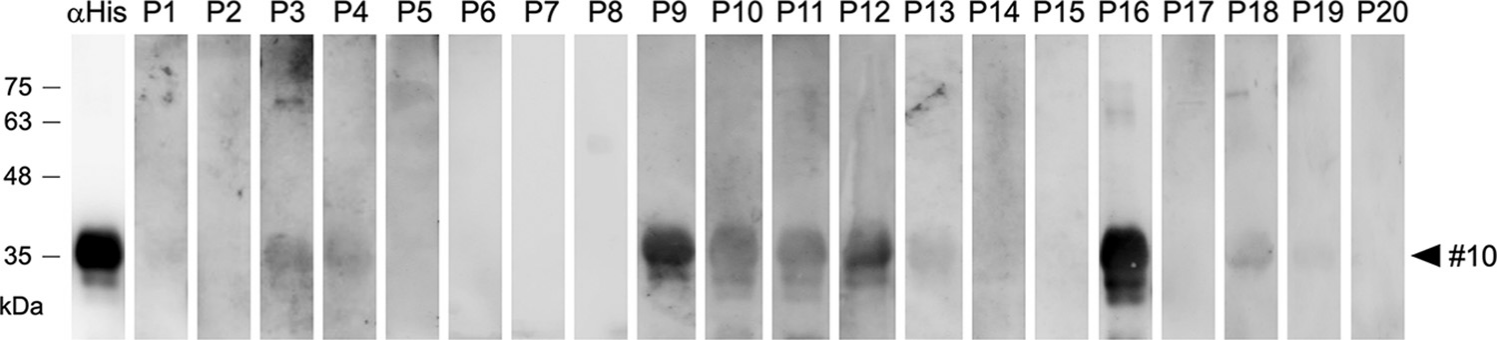
The human sera from COVID-19 patients contain S(1127-1167)-specific antibodies. The sfGFP-S(1127-1167)-His (#10) recombinant proteins (2 μg) were separated on SDS-PAGE and subjected to Western blot analysis with anti-His tag MAb (1:2,000) and the human sera (1:1,000) randomly collected from 20 hospitalized COVID-19 patients (P1-P20) within 14–21 days since they were diagnosed with SARS-CoV-2 infection by RT-qPCR.

Although evidence on the possibility of inducing neutralizing antibodies via immunization of mice with sfGFP-S(1127-1167) was demonstrated, it’s unclear if the human immune system is capable of eliciting such specific antibodies. To clarify this, the human sera randomly collected from 20 hospitalized COVID-19 patients within 14–21 days since they were first diagnosed with SARS-CoV-2 infection by RT-qPCR were subjected to Western blot analysis. The results showed that the human sera from some COVID-19 patients (such as sera from Patient 9 and Patient 16) indeed contain antibodies which can bind to the S(1127-1167) epitope peptide and exhibit strong Western blot signal (Fig. 5), indicating that the specific antibodies against S(1127-1167) epitope peptide can be elicited through natural SARS-CoV-2 infection in humans.

### Neutralization of diverse SARS-CoV-2 variants by S2-4D, S2-5D, and S2-8D.

**FIG 6:**
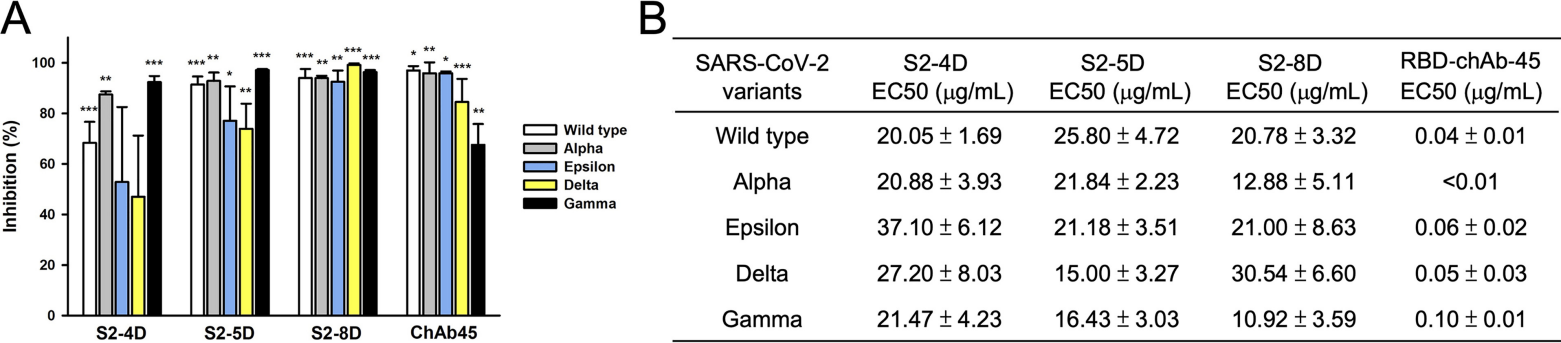
Neutralization of SARS-CoV-2 Alpha, Gamma, Delta, and Epsilon variants by mAbs S2-4D, S2-5D, and S2-8D. (A) The mAbs S2-4D, S2-5D, S2-8D (60 μg/mL), and a neutralizing chimeric antibody RBD-chAb-45 (100 ng/mL) against the RBD of SARS-CoV-2 S protein were preincubated with SARS-CoV-2 wild type (white bars), Alpha (gray bars), Epsilon (blue bars), Delta (yellow bars), and Gamma (black bars) variants and then subjected to the plaque reduction assay. Five to 7 days later, plaques were counted and the inhibition of the plaque formation was calculated by comparing to the experimental results in the absence of antibodies. Data are presented as means ± SD of three biological replicates (*n* =3). One-way ANOVA was used for statistical analysis. *, *P ≤ *0.05; **, *P ≤ *0.01; ***, *P ≤ *0.001. (B) The EC50 values of S2-4D, S2-5D, S2-8D, and RBD-chAb-45 against different SARS-CoV-2 variants.

A reduced neutralization activity of therapeutic mAbs or sera from convalescent patients against the SARS-CoV-2 variants carrying genetic mutations in the S proteins, such as SARS-CoV-2 Alpha and Beta variants, has been reported (32). However, a neutralizing chimeric antibody RBD-chAb-45 against the RBD of SARS-CoV-2 S protein exhibited strong neutralizing potency against SARS-CoV-2 D614G, Alpha, Beta, Gamma, Delta, Epsilon, Iota, and Kappa variants (33). Therefore, the ability of S2-4D, S2-5D, and S2-8D to neutralize SARS-CoV-2 Alpha, Gamma, Delta, and Epsilon variants was examined by plaque reduction assay. As shown in Fig. 6A, a significant inhibition of virus infection was observed for S2-4D, S2-5D, S2-8D, and RBD-chAb-45 against all of the SARS-CoV-2 wild type (white bars), Alpha (gray bars), Epsilon (blue bars), Delta (yellow bars), and Gamma (black bars) variants, revealing that S2-4D, S2-5D, S2-8D, and RBD-chAb-45 exhibited neutralizing activity against a broad range of SARS-CoV-2 variants although the neutralizing activity of S2-4D against SARS-CoV-2 Gamma and Delta variants was not statistically significant. For determination of the EC50 values of S2-4D, S2-5D, S2-8D, and RBD-chAb-45 against different SARS-CoV-2 variants, a series of diluted S2-4D, S2-5D, S2-8D, and RBD-chAb-45 solutions were utilized in the plaque reduction assay (Fig. S2). The results showed that the EC50 values of S2-4D, S2-5D, and S2-8D against Alpha, Gamma, Delta, and Epsilon are in the ranges of 10–40 μg/mL (Fig. 6B). S2-5D has the lowest EC50 value for neutralizing the Delta variant. S2-8D has the lowest EC50 values for neutralizing the Alpha and Gamma variants. In contrast, RBD-chAb-45 exhibited stronger neutralizing potency against all of the variants, with very low EC50 values ranging from 40 to 100 ng/mL (Fig. 6B).

### Inhibition of S protein-mediated membrane fusion and SARS-CoV-2 infection by S2-4A.

**FIG 7:**
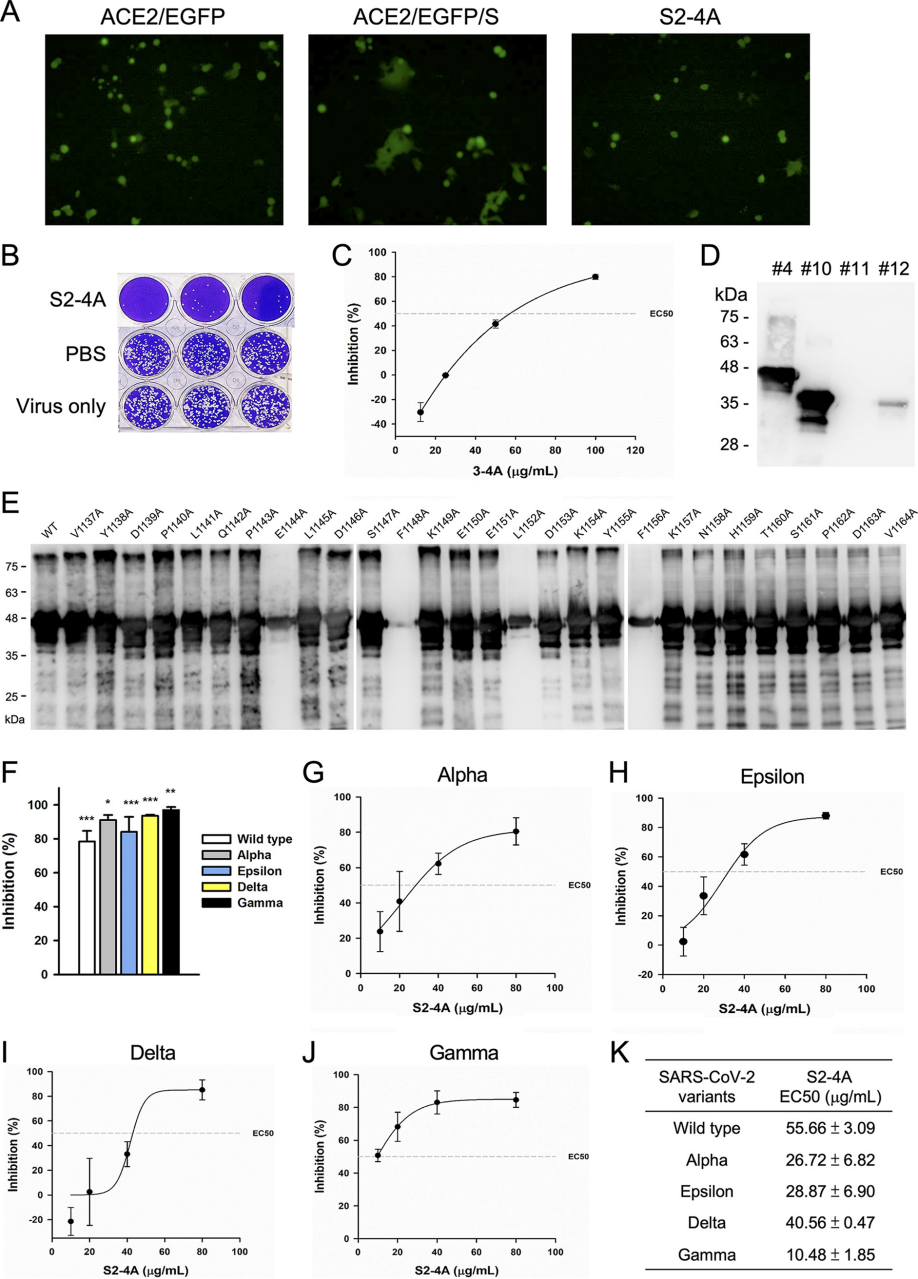
The virus neutralizing activity and antigenic specificity of MAb S2-4A. (A) The S protein-mediated syncytium formation assay was performed to analyze the neutralizing activity of MAb S2-4A. The formation of the syncytia by coculturing the EGFP-transfected ACE2-293T cells with the S protein-transfected 293T cells was markedly inhibited in the presence of S2-4A (100 μg/mL). (B) The MAb S2-4A (100 μg/mL) was incubated with the SARS-CoV-2 wild-type strain. and the plaque reduction assay was applied to evaluate the neutralizing activity. Data are three biological replicates (*n* = 3). (C) The EC50 of MAb S2-4A against SARS-CoV-2 was determined by using a series of MAb S2-4A (100, 50, 25, 12, 5 μg/mL) in the plaque reduction assay. (D) The binding of MAb S2-4A to S2 truncation fragments #4, #10, #11, and #12 was determined by WB analysis. (E) The sfGFP-S(1042-1167) mutants with a series of individual alanine substitution at residues 1137–1164 were analyzed on SDS-PAGE and then subjected to WB analysis with MAb S2-4A to identify the critical antigenic determinants. (F) The S2-4A (100 μg/mL) was preincubated with the SARS-CoV-2 wild-type strain (white bar), Alpha (gray bar), Epsilon (blue bars), Delta (yellow bars), and Gamma (black bars) variants and then subjected to the plaque reduction assay. The inhibition of the plaque formation by MAb S2-4A was calculated by comparing to the experimental results in the absence of antibodies. Data are presented as means ± SD of three biological replicates (*n* = 3). One-way ANOVA was used for statistical analysis. *, *P ≤ *0.05; **, *P ≤ *0.01; ***, *P ≤ *0.001. (G–J) For determination of the EC50 values of S2-4A against SARS-CoV-2 Alpha, Epsilon, Delta, and Gamma variants, a series of diluted MAb S2-4A solutions (80, 40, 20, and 10 μg/mL) were utilized in the plaque reduction assay. Data are presented as means ± SD of three biological replicates (*n* =3), and further graphed by linear regression. (K) The EC50 values of S2-4A against different SARS-CoV-2 variants.

It is worth mentioning that S2-4D, S2-5D, and S2-8D were obtained from three different hybridoma cell lines, but they all recognized the same antigenic determinants, suggesting that they were induced by the same immunodominant epitope. In addition, S(1127-1167) has been demonstrated to be an immunogenic peptide that can induce nAbs (Fig. 4). Therefore, we tried to perform another attempt of MAb production beginning from immunization of mice with the S2 subunit. As a result, we obtained a MAb S2-4A that can also inhibit the S protein-mediated membrane fusion (Fig. 7A) and block SARS-CoV-2 infection in Vero E6 cells (Fig. 7B) with an EC50 value of 60 μg/mL (Fig. 7C). Surprisingly, S2-4A exhibited similar antigenic specificity as that of S2-4D, S2-5D, and S2-8D for binding to fragments #4 and #10 (Fig. 7D, lanes 1 and 2). However, S2-4A performed a stronger binding reactivity with the fragment #12 (Fig. 7D, lane 4), which is not well recognized by S2-4D, S2-5D, and S2-8D, implying that the antigenic determinants of S2-4A might be slightly different. The detailed epitope mapping results demonstrated that, in addition to the residues E1144, F1148, and F1156, residue L1152 was also important for epitope recognition by S2-4A (Fig. 7E). These findings suggested that the 13-residue peptide, S(1144-1156), contains a unique immunodominant and neutralizing epitope. Moreover, S2-4A also exhibited broad-spectrum neutralizing activity against SARS-CoV-2 Alpha, Gamma, Delta, and Epsilon variants (Fig. 7F), with EC50 values ranging from 10 to 55 μg/mL, respectively (Fig. 7G to K).

### Mapping of the antigenic determinants E1144, F1148, L1152, and F1156 on the prefusion and postfusion structures of the trimeric S protein and S2 subunit.

**FIG 8:**
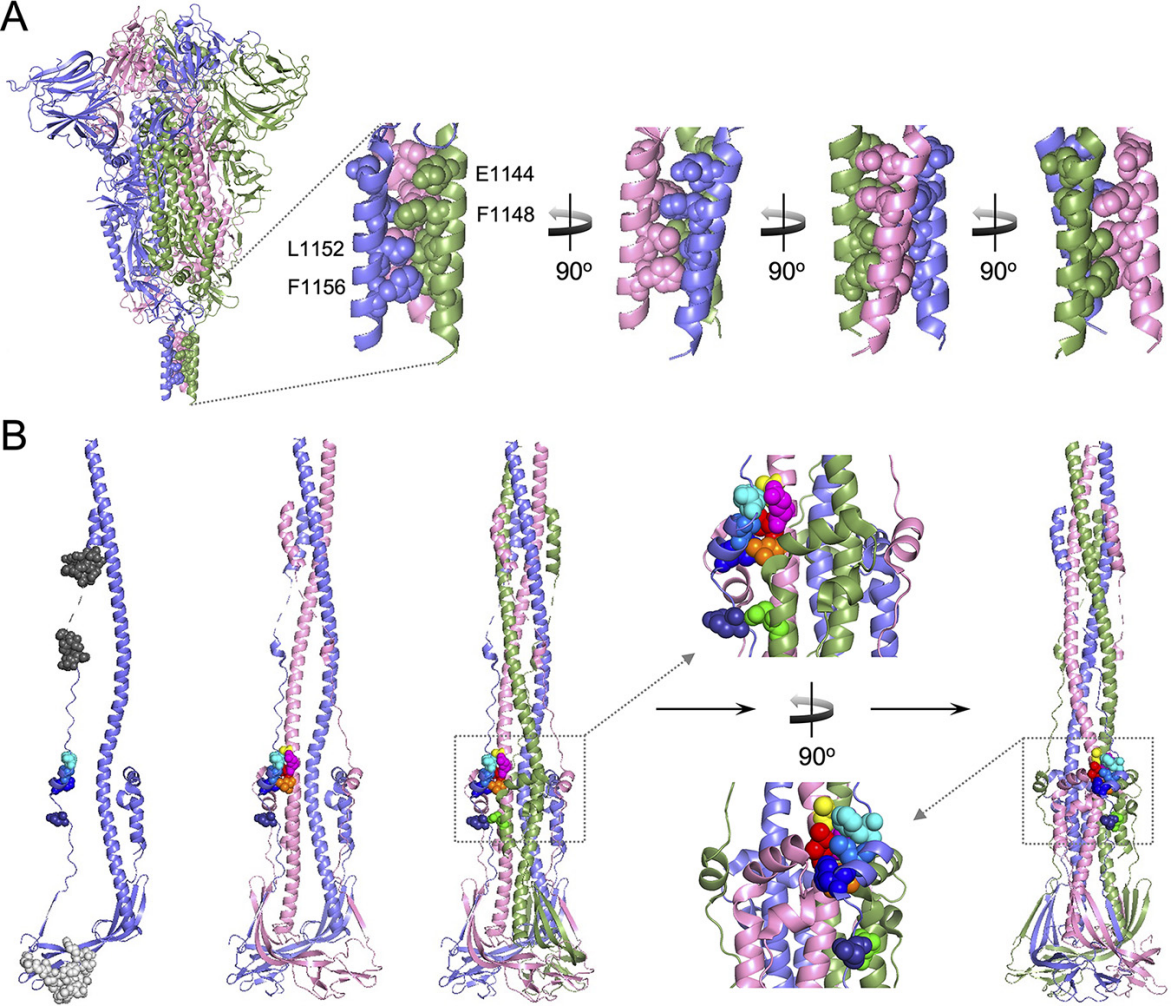
Mapping of the antigenic determinants recognized by S2-4D, S2-5D, S2-8D, and S2-4A on the prefusion and postfusion structures of the trimeric S protein and S2 subunit. (A) The antigenic determinants (E1144, F1148, L1152, F1156) of mAbs S2-4D, S2-5D, S2-8D, and S2-4A were mapped on the prefusion structure of the trimeric full-length S protein (PDB code: 6xr8). The carboxyl side chain groups of E1144 are pointed outwards, and residues F1148, L1152, and F1156 are buried inside a triple-helical structure and form a hydrophobic core. (B) The binding epitopes of mAbs S2-10H and S2-8A are marked in light gray spheres and dark gray spheres, respectively. Residues E1144 (deep blue), F1148 (blue), L1152 (marine blue), and F1156 (cyan) are located at the peripheral part of a six-helix bundle on the stalk-like structure of the S2 subunit (PDB code: 6xra). The residue E1144 of Subunit A (light blue) interacts with the residue Q762 (green) of the adjacent Subunit C (light green), and F1148, L1152, and F1156 of Subunit A interacts with the residues A989 (yellow), Q992 (magenta), I993 (red), and L996 (orange) of another adjacent Subunit B (pink).

Cryo-electron microscopy (cryo-EM) structures of the prefusion and postfusion states of the trimeric full-length S protein of SARS-CoV-2 have been solved (34). The localization of E1144, F1148, L1152, and F1156 was mapped on the prefusion structure of the S trimer (PDB codes: 6xr8) or the postfusion structure of the S2 trimer (PDF codes: 6xra) (Fig. 8). In the prefusion state, E1144, F1148, L1152, and F1156 are located in a triple-helical structure at the bottom of the S trimer (Fig. 8A). The carboxyl side chain groups of three E1144 residues are pointed outwards, but the hydrophobic side chain groups of F1148, F1152, and F1156 from three subunits are thoroughly buried inside the triple-helical structure and form a hydrophobic core (Fig. 8A). In the postfusion state, since the S2 subunit undergoes dramatic conformational changes, F1148, L1152, and F1156 (Fig. 8B, Subunit A) start interacting with a hydrophobic patch composed of A989, Q992, I993, and L996 from an adjacent subunit (Fig. 8B, Subunit B). E1144 (Fig. 8B, Subunit A) starts interacting with Q762 via a hydrogen bond from another adjacent subunit (Fig. 8B, Subunit C). Because the critical antigenic determinants recognized by S2-4D, S2-5D, S2-8D, and S2-4A are located at the immediately upstream region of HR2 (Fig. S3), which is predicted to be located within residues 1162–1205 (35) or 1163–1211 of the SARS-CoV-2 S protein (34), we propose that binding of S2-4D, S2-5D, S2-8D, and S2-4A to this region can prevent the S2 subunit from forming a six-helix bundle core of HR1 and HR2, leading to blockage of membrane fusion and virus infection. Notably, S2-8A cannot inhibit S protein-mediated membrane fusion and SARS-CoV-2 infection (Fig. 2A and B) even though its binding epitope is located at residues 1168–1187 within the HR2 domain (Fig. 3G). Unfortunately, the residues 1168–1187 were not observed in the prefusion state of S protein and the residues 1174–1179 were not visible in the postfusion state of the S2 subunit in the published structures (Fig. 8B) (34). Thus, it is concluded that the binding epitope of S2-8A may not play a key role in the HR1-HR2 six-helix bundle formation, or binding of S2-8A to its epitope cannot sterically interrupt the conformational change of the S2 subunit. In the postfusion state of the S2 subunit, the binding epitope of S2-10H is located at the basal region of the stalk-like S2 subunit near the viral membrane (Fig. 8B). The accessibility for S2-10H binding to its epitope might be sterically hindered, therefore S2-10H could not inhibit the S protein-mediated membrane fusion and SARS-CoV-2 infection (Fig. 2A and B).

## DISCUSSION

The neutralizing activity of the anti-RBD antibody is largely reduced while the immune escape sequence mutations of SARS-CoV-2 variants are located at the RBD of the S proteins (32, 36). Nevertheless, an antibody targeting the relatively conserved S2 subunit of the S protein might provide broadly neutralizing activity against diverse SARS-CoV-2 variants. The present study demonstrated that mAbs S2-4D, S2-5D, S2-8D, and S2-4A, which were generated by immunization of mice with the S2 subunit, recognized a highly conserved 13-residue epitope found in the S proteins of SARS-CoV-2, SARS-CoV, and MERS-CoV (Fig. S3). More importantly, S2-4D, S2-5D, S2-8D, S2-4A and the antisera obtained by immunization of mice with sfGFP-S(1127-1167) (Fig. 4), which carries the defined 13-residue epitope, can inhibit the S protein-mediated membrane fusion and SARS-CoV-2 infection, indicating that these antibodies neutralized virus infection through blocking the key step of virus–host cell membrane fusion. Interestingly, S(1127-1167) can be recognized by S2-4D, S2-5D, S2-8D, and S2-4A, but the individual S(1127-1146) or S(1147-1167) cannot be properly recognized, suggesting that both of the regions near the C-terminus of S(1127-1146) and the N-terminus of S(1147-1167) contain the critical residues for antibody recognition. The approach using the site-directed mutagenesis with alanine substitution further demonstrated that E1144, F1148, L1152, and F1156 are the key antigenic determinants for S2-4D, S2-5D, S2-8D, and S2-4A binding (Fig. 3K-N and Fig. 7E).

The evidence for the possibility to induce neutralizing antibodies via immunization of mice with sfGFP-S(1127-1167) was demonstrated (Fig. 4). The significance of the S(1127-1167) epitope peptide was further confirmed by the Western blot analysis using the human sera collected from COVID-19 patients (Fig. 5), indicating that natural SARS-CoV-2 infection could also elicit S2-4D, S2-5D, S2-8D, and S2-4A-like antibodies through natural SARS-CoV-2 infection. In the study reported by Jennewein et al. (37), only one MAb CV3-25 out of 87 anti-S2 mAbs among 198 antibodies isolated from four COVID-19 patients was capable of neutralizing SARS-CoV-2 and SARS-CoV and of binding the S proteins of the human betacoronaviruses OC43 and HKU1. This result suggested that CV3-25 binds a unique epitope on the S2 subunit, which is conserved not only on SARS-CoV-2 and SARS-CoV, but also on the other coronaviruses. Since CV3-25 does not interfere with the binding of ACE2 to S protein, it is anticipated that CV3-25 mediates neutralization by interfering with a step in the fusion process that follows attachment. Although the epitope of CV3-25 was not yet identified, their findings and our results demonstrated that the S2 subunit contains at least one epitope that is a valid candidate for the development of a broad-spectrum coronavirus vaccine.

The HR2 domain of the SARS-CoV S protein has been predicted to be located within residues 1125–1193 (38) or 1144–1191 (39). One human scFv, B1, which was selected from an immune antibody phage-display library constructed from B cells of SARS-CoV infected convalescent patients, showed potent neutralizing activities against pseudoviruses carrying the SARS-CoV S proteins *in vitro*, and recognized an epitope within amino acid residues 1023–1189 of the S2 subunit (27). It has also been reported that the amino acid residues 1055–1192 in the S2 subunit of SARS-CoV S protein can induce neutralizing antibodies (28). The mAbs raised against residues 1055–1192 showed neutralizing activities (29). The MAb 1A9 targeting an upstream linker region (residues 1111–1130) of HR2 of SARS-CoV S protein was able to neutralize virus infection with an IC50 of 35 μg/mL. Furthermore, MAb 1A9 exhibited cross-protection against viral entry mediated by the S proteins of human SARS-CoV, civet SARS-CoV, and bat SL-CoV (29). SARS-CoV S protein containing the D1128A mutation exhibited a significant decrease in binding capability to MAb 1A9, and D1128A substitution enables the virus to escape inhibition by MAb 1A9 (40). The cryo-EM analysis of the postfusion structure of the SARS-CoV S protein has been reported recently (8). Note that D1128 and its adjacent residues 1111–1130 within the upstream linker region of HR2 are highly conserved between SARS-CoV and SARS-CoV-2 (Fig. S3). MAb 1A9 can also detect the SARS-CoV-2 S protein (41). Similarly, we also showed that S2-4D, 5D, and S2-8D performed cross-reactivity with SARS-CoV S protein (Fig. 1).

It has been observed that the neutralizing titers of the mAbs against membrane fusion domains were relatively lower compared with that of mAbs targeting the RBD of SARS-CoV S protein (29, 42, 43). Previous findings on murine coronavirus also showed that the neutralizing titers of mAbs against the S2 subunit are 3 to 4 orders of magnitude lower than those against RBD (44, 45). We also observed that S2-4D, S2-5D, S2-8D, and S2-4A exhibited neutralizing activities, with EC50 values ranging from 20 to 60 μg/mL (Fig. 2), which were indeed markedly lower than the ng/mL values of the RBD-chAb-45 (Fig. 6B) and the human mAbs against the RBD of SRAS-CoV-2 S protein (15, 17, 46). We also found that S2-4D exhibited weaker neutralizing potency against all of the variants than did S2-5D (Fig. 6A). Because the binding epitopes of S2-4D and S2-5D are identical in these variants tested here, we think that the possible reasons for differential neutralization capability between S2-4D and S2-5D is that the S2-4D is less resistant to trypsin treatment in the plaque reduction assay; therefore, the weaker neutralizing activity of S2-4D were subsequently observed. Despite the broadly neutralizing activity, how to improve the potency and stability of anti-S2 nAb remains a challenge for the future development of therapeutics.

It has been known or predicted that some genetic mutations of SARS-CoV-2 may affect virus characteristics such as increase in transmissibility, disease severity, and immune escape, or decrease in effectiveness of available diagnostics, vaccines, and therapeutic medicines, resulting in an emerging risk to global public health (47–49). Human mAbs that bind to conserved regions of the S protein were considered more suitable for conferring protection against a wide range of SARS-CoV variants (50, 51). As SARS-CoV-2 continues to spread worldwide, population-level immunity elicited by natural infection may induce the immune escape mutants under the selective pressure of nAbs. Several critical mutations in the RBD have been observed in diverse SARS-CoV-2 variants, resulting in reduction or loss of the neutralizing activity of the RBD-specific antibodies. In contrast, the upstream region of HR2 in the S2 subunit is still completely conserved in different SARS-CoV-2 isolates, indicating that the fusion mechanism during virus infection is essential and well-conserved. We believe that mAbs S2-4D, S2-5D, S2-8D, and S2-4A with virus-neutralizing activity against the diverse and highly transmissible SARS-CoV-2 variants are promising candidates for developing as therapeutics to prevent or treat COVID-19. The binding epitopes of S2-4D, S2-5D, S2-8D, and S2-4A are the important targets for designing a broad-spectrum vaccine against the emerging SARS-like coronaviruses.

## MATERIALS AND METHODS

### Expression of the recombinant S, NTD, RBD, and S2 proteins.

The cDNA encoding for the amino acid residues 16–1208 of SARS-CoV-2 S (GenBank: BCN86353.1) with R682A, R683A, and R685A mutations, residues 16–310 of SARS-CoV-2 S (NTD), residues 331–520 of SARS-CoV-2 S (RBD), or residues 686–1208 was PCR-amplified from a mammalian codon-optimized S gene sequence (GenScript, Cat. No. MC_0101081), and then subcloned into the pSectag2A vector (Thermo Fisher Scientific) with an N-terminal secretion signal and a C-terminal hexa-histidine tag (His-tag). The recombinant proteins were expressed by using the Expi293 Expression System (Thermo Fisher Scientific) according to the manufacturer’s instruction with slight modifications. Briefly, Expi293F cells (4 × 10^6^ cell/mL) were cultured in Expi293F Expression medium (Thermo Fisher Scientific) in a 37°C incubator with 80% humidity and 8% CO_2_ on an orbital shaker and transfected with the constructs using the ExpiFectamine 293 Reagent (Thermo Fisher Scientific) or polyethylenimine (PEI, Polysciences). Twenty hours posttransfection, 2 mM sodium butyrate (Sigma-Aldrich) was added to the medium and cultured for up to 7 days. Culture medium was collected by centrifugation at 4,000 × *g* for 30 min, and the supernatant was filtered through a 0.22 μm bottle-top filter. The His-tagged protein was purified using the Ni-NTA resin (MAM-50 His_NTA resin, EBL Biotechnology) with the binding buffer (20 mM NaH_2_PO_4_, 0.5 M NaCl, 5 mM imidazole, pH 7.4) and the elution buffer (20 mM NaH_2_PO_4_, 0.5 M NaCl, 0.5 M imidazole, pH 7.4). Buffer exchange of the purified His-tagged protein solution with phosphate-buffered saline (PBS) was then performed by using the PD-10 desalting column (GE Healthcare). The protein concentration was determined by the Bradford dye-binding method (52).

### Preparation of mouse mAbs.

The animal experiments were approved by the Institutional Animal Care and Use Committee of National Taiwan University (approval number: NTU-109-EL-00051) and implemented in accordance with the animal care and ethics guidelines. The procedures for generation of hybridomas were performed as described previously (53, 54) with slight modifications. In brief, two BALB/c male mice were immunized one time with a mixture of S2 (100 μg) and complete Freund’s adjuvant (0.25 mL) and two times with a mixture of S2 (50 μg) and incomplete Freund’s adjuvant (0.25 mL) at a 2-week interval through intraperitoneal injection, followed by a final booster injection of S2 (50 μg) in 0.25 mL of PBS. The Sp2/0-Ag14 myeloma cells were fused with splenocytes derived from the donor mouse immunized with S2 in the presence of 0.7 mL of polyethylene glycol 1500 (Sigma-Aldrich) at 37°C for 2 min with gentle shaking, and 10 mL of Dulbecco’s Modified Eagle Medium (DMEM) was then added to the cell mixture. Cells were collected by centrifugation and resuspended in 30 mL of DMEM with 15% fetal bovine serum (FBS), 1% penicillin-streptomycin, 1 mM sodium pyruvate (Thermo Fisher Scientific), and HAT media supplement (Sigma-Aldrich). Fusion cells were then cultured in the 96-well plates at 37°C in the 5% CO_2_ incubator. The culture media were collected to screen for the positive hybridoma clones by enzyme-linked immunosorbent assay (ELISA) using the S protein as the antigen. The mAbs were further selected by limiting dilution method. To purify the mAbs, the hybridoma cell culture media were filtered through a 0.45 μm membrane disc and then purified by HiTrap Protein G HP column (GE Healthcare). The purified antibody was dissolved in PBS and the concentration was determined by Bradford dye-binding method using mouse IgG as the standard.

### ELISA.

The wells of a 96-well plate precoated with 100 ng of NTD, RBD, S2, and S proteins of SARS-CoV-2, SARS-CoV (Sino Biological Inc., Catalog Number: 40634-V08B) or MERS-CoV (Sino Biological Inc., Catalog Number: 40069-V08B) were blocked with 0.25% gelatin in PBS containing 0.05% Tween 20 (PBST), and then incubated with 100 μL of antibody solution at room temperature for 1 h. After washing of the 96-well plate three times with PBST, the HRP-conjugated goat antimouse IgG (H+L) (KPL) (1:4,000 in PBST) was added to each well and incubated at room temperature for another 1 h. After washing of the 96-well plate three times with PBST, 75 μL of 3,3′,5,5′-tetramethylbenzidine substrate (BD Bioscience) was added to each well for 5 min at room temperature before adding 75 μL 2 N H_2_SO_4_ to terminate the reactions. Results are evaluated quantitatively by measuring the absorbance at 450 nm by the Multiskan FC Microplate Photometer (Thermo Fisher Scientific).

### ACE2-transfected stable cell line.

The human ACE2 mammalian expression vector pCMV3-C-His-hACE2 (GenBank: NM_021804.1) was purchased from Sino Biological Inc. (Catalog Number: HG10108-CH), and then transfected to human embryonic kidney 293T (293T) cells by using the Lipofectamine 3000 reagent (Invitrogen, USA) according to the manufacturer’s instruction. Twenty-four hours after transfection, 150 μg/mL of hygromycin (InvivoGen, USA) was added to the culture medium for selection of the ACE2-expressed 293T stable cell line (named as ACE2-293T).

### S protein-mediated syncytium formation assay.

The 293T cells transfected with the pcDNA3.1-S gene plasmid were used as the effector cells, and ACE2-293T cells transfected with the pcDNA3-EGFP plasmid were used as the target cells. Effector cells and target cells were cocultured in DMEM containing 10% FBS to trigger the S protein-mediated membrane fusion. To determine whether the mAbs or antisera could block S protein-mediated cell fusion, mAbs or antisera were preincubated with effector cells for 1 h at 37°C, and then added to the monolayered target cells in a 96-well plate. After 16 h of incubation, the syncytium formation was observed by fluorescence microscopy (AE31E Trinocular live cell microscope, Motic).

### Plaque reduction assay.

All experiments related to SARS-CoV-2 viruses were conducted in the BSL-3 laboratory. Antibody solution or antiserum was incubated with 150 PFU of SARS-CoV-2 wild-type strain (EPI_ISL_422415; A.3), Alpha (EPI_ISL_1010728; B.1.1.7), Gamma (EPI_ISL_2249499; P.1), Delta (EPI_ISL_3979387; B.1.617.2), or Epsilon (EPI_ISL_1020315; B.1.429) variant in the presence of 8 μg/mL TPCK-trypsin of DMEM for 1 h at 37°C. Antibody–virus mixtures (200 μL/well) were subsequently added to the Vero E6 cell monolayers, which were pretreated with 2 μg/mL TPCK-trypsin in the 24-well plates. After 1 h, cells were washed with PBS and overlaid with 2% (wt/vol) methylcellulose in DMEM supplemented with 2% FBS, and then incubated at 37°C in the 5% CO_2_ incubator. Five to 7 days later, cells were fixed with 10% formaldehyde for 1 h and stained with 0.5% crystal violet. After that, plaques were counted and the 50% effective concentration (EC50) of the antibody was determined by linear regression analysis.

### Expression of S2 truncation fragments.

The cDNAs encoding for S2 truncation fragments utilized in the study were subcloned into the pET30a vector (Novagen, Merck Group) with a C-terminal His-tag or an additional N-terminal superfolder GFP (sfGFP) fusion protein (31). The bacterial expression vectors were transformed into E. coli BL21(DE3) competent cells and cultured in Luria-Bertani medium with kanamycin (50 μg/mL) at 37°C on an orbital shaker. Protein expression was induced at an A_600_ of 0.4–0.6 by adding isopropyl-1-thio-beta-d-galactopyranoside to a final concentration of 1 mM for 4 h. Cells were collected by centrifugation at 8,000 × *g* for 10 min, and the pellet was homogenized by cell disruptor (Constant Systems Limited, UK) in buffer A (20 mM sodium phosphate, 20 mM imidazole, 0.5 M NaCl, pH 7.4). The His-tagged proteins were purified by HisTrap FF column (GE Healthcare), and the bound proteins were eluted with a 20–500 mM gradient of imidazole in buffer A. Buffer exchange of the purified His-tagged proteins with PBS were then performed by using the PD-10 desalting column (GE Healthcare). The protein concentration was determined by the Bradford dye-binding method.

### Preparation of mouse antisera.

The primary immunization was performed through intraperitoneal injection of 50 μg sfGFP-fusion proteins mixed with complete Freund’s adjuvant. Mice were subsequently boosted with 25 μg of the sfGFP-fusion proteins in incomplete Freund’s adjuvant at week 2 and week 4. The final immunization was boosted with 25 μg of the sfGFP-fusion proteins in PBS at week 6. The antisera were collected, heated at 56°C for 30 min, filtered through a 0.22 μm membrane disc, and then utilized for WB analysis, S protein-mediated syncytium formation assay, and plaque reduction assay.

### Site-directed mutagenesis.

The pET30a-sfGFP-S(1042-1167)-His was used as the template for site-directed mutagenesis. The codons encoding for residues 1137–1164 were substituted individually with the codons of alanine by using the PCR-based site-directed mutagenesis method. All mutations were confirmed by Sanger sequencing. The vectors for expressing the sfGFP-S(1042-1167)-His mutants were transformed into E. coli BL21(DE3) competent cells, and the protein expressions were induced as described previously. Bacterial cells were collected for protein extraction, which was then subjected to SDS-PAGE and WB analysis with the indicated antibodies.

### Human sera.

Human sera were randomly collected from 20 hospitalized COVID-19 patients within 14–21 days since they were first diagnosed with SARS-CoV-2 infection by RT-qPCR. The collected sera were heated at 56°C for 30 min, and then stored at 4°C. A written informed consent was obtained from all individual patients in the study, which was approved by the Institutional Review Board of National Taiwan University Hospital.

### Statistical analysis.

Statistical analyses were performed with SigmaPlot version 12.5 (Systat Software, Inc., Chicago, IL, USA). The statistical significance was calculated with one-way ANOVA. *, *P ≤ *0.05; **, *P ≤ *0.01; ***, *P ≤ *0.001.

### Data availability.

All data generated in this study are included in this published article and in the supplemental material.
