# Supplementary material for: Neutralizing Monoclonal Antibodies Inhibit SARS-CoV-2 Infection through Blocking Membrane Fusion
Source: Microbiol Spectr. 2022 Mar 16;10(2):e01814-21. doi: 10.1128/spectrum.01814-21 (PMC9045258; doi:10.1128/spectrum.01814-21)
Supplement: SUPPLEMENTAL FILE 1 — Supplemental material. Download SPECTRUM01814-21_Supp_1_seq12.pdf, PDF file, 1.4 MB [file spectrum01814-21_supp_1_seq12.pdf]

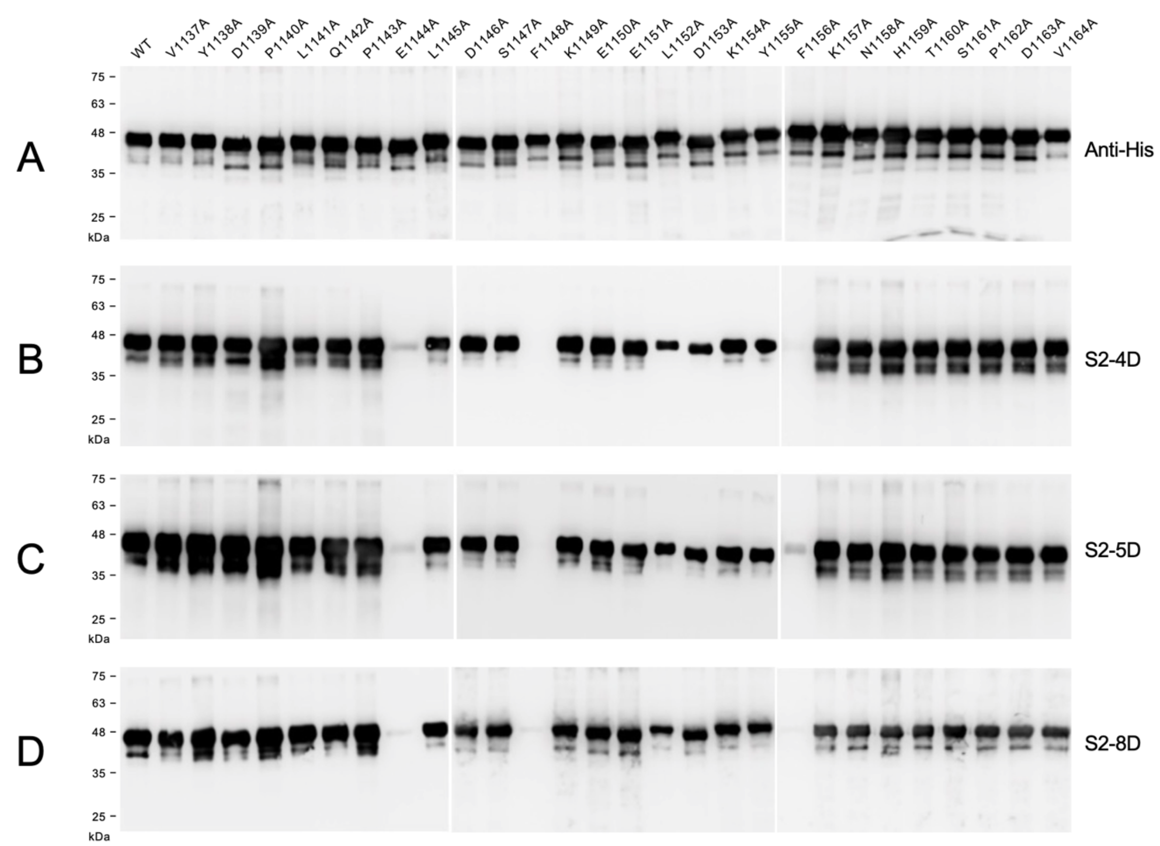

FIG S1 Analysis of antigenic determinants of mAbs S2-4D, S2-5D, and S2-8D. The ability of the control anti-His tag antibody (A), S2-4D (B), S2-5D (C), or S2-8D (D) to recognize sfGFP-S(1042-1167) mutants with a series of individual alanine substitution at residues 1137-1164 were determined by WB analysis using 2 μg of bacterial cell lysates on the SDS-PAGE.

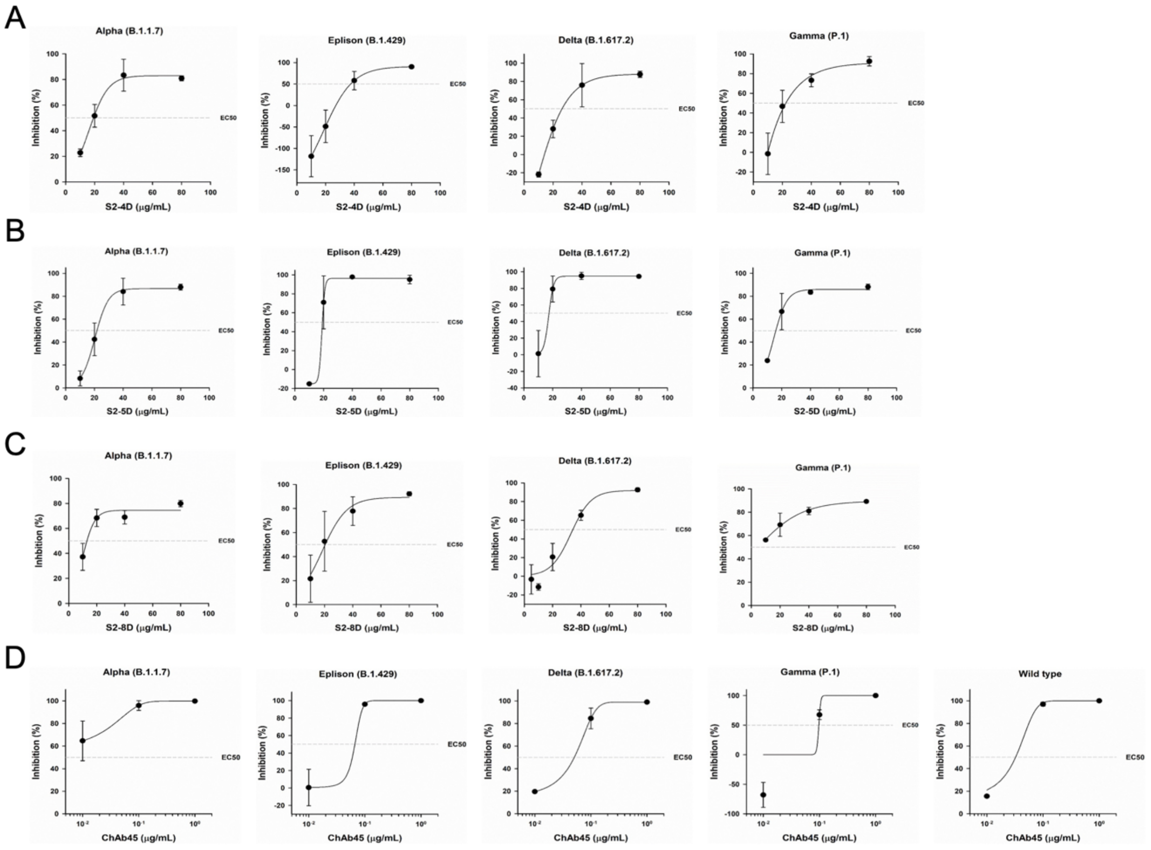

FIG S2 Determination of the EC50 values of S2-4D, S2-5D, S2-8D, and RBD-chAb-45 against different SARS-CoV-2 variants. (A-D) For determination of the EC50 values of S2-4D, S2-5D, S2-8D, and RBD-chAb-45 against SARS-CoV-2 wild type, Alpha (B.1.1.7), Epsilon (B.1.429), Delta (B.1.617.2), and Gamma (P.1) variants, a series of diluted S2-4D, S2-5D, S2-8D (80, 40, 20, and 10 μg/mL), and RBD-chAb-45 solutions (1, 0.1, and 0.01 μg/mL) were utilized in the plaque reduction assay. Data are presented as means ± SD of three biological replicates (n =3), and further graphed by linear regression.

```
              1109                1129                1149
               |                   |                   |
SARS-CoV      DVVIGIINNTVYDPLQPELDSFKGELDKYFKNHTSPDVDLG   HR2
SARS-CoV-2    DVVIGIVNNTVYDPLQPELDSFKEELDKYFKNHTSPDVDLG
              ******:*************** ****************
               |                   |                   |
              1127                1147                1167
```

FIG S3 Sequence alignment of the binding epitopes of mAbs 1A9, S2-4D, S2-5D, S2-8D, and S2-4A in S proteins of SARS-CoV and SARS-CoV-2. The amino acid residues 1109-1149 of SARS-CoV S protein (GenBank: AAR86775.1) and the corresponding amino acid residues 1127-1167 of SARS-CoV-2 S protein (GenBank: BCN86353.1) were aligned. The identical residues were marked with asterisks. The binding epitope of mAb 1A9 was marked with a light gray background. The binding epitope of S2-4D, S2-5D, S2-8D, and S2-4A was marked with a dark gray background. The residue D1128 in SARS-CoV S protein and residues E1144, F1148, L1152, and F1156 in SARS-CoV-2 S protein were noted in bold characters. The partial region of the predicted HR2 domain was labeled in a square box.
